# Impact of Ambient Artificial Intelligence Documentation on Cognitive Load

**DOI:** 10.1016/j.mcpdig.2024.100193

**Published:** 2025-01-02

**Authors:** Taina J. Hudson, Michael Albrecht, Timothy R. Smith, Gregory A. Ator, Jeffrey A. Thompson, Tina Shah, Denton Shanks

**Affiliations:** a University of Kansas Medical Center, Kansas City, KS; b RWJ Barnabas Health Newark Beth Israel Medical Center, Newark, NJ; c Abridge AI, Inc, Pittsburgh, PA

Despite significant advancements since the Institute of Medicine’s 1999 report, *To Err is Human*, which highlighted that preventable medical errors contributed to up to 98,000 hospital deaths annually,^1^ reducing preventable harm remains a critical challenge. A central factor contributing to medical errors is the high cognitive load experienced by clinicians, arising from the need to process a vast volume of complex patient data while fulfilling excessive administrative and regulatory requirements with clinical documentation. The lack of effective tools for managing these tasks leads to increased mental fatigue among providers, negatively impacting clinician well-being, diverting focus from direct patient care, and putting patient safety at risk.^2^^,^^3^

Artificial intelligence (AI) tools offer promising avenues to alleviate clinician cognitive load. Specifically, ambient AI documentation platforms may streamline clinical workflows by efficiently capturing and organizing clinical information, thus reducing the mental effort required by clinicians. However, research on AI’s effectiveness in reducing cognitive load and its impact on medical errors and provider burnout remains limited.^4^ This study evaluated the effect of an ambient AI documentation platform on clinician cognitive load.

## Methods

We recruited 40 ambulatory providers at a large academic institution through convenience sampling for a prospective interventional study from March 2024 to April 2024. Participants selected 2 comparable clinic sessions and were randomized to first use either Abridge—an electronic health record-integrated ambient AI platform—or their usual note-writing process, then switched to the alternate method during the second session.

We assessed cognitive load using 3 dimensions of the validated National Aeronautics and Space Administration Task Load Index (NASA-TLX): effort, mental demand, and temporal demand, selected for their relevance to documentation tasks.^3^^,^^5^ The NASA-TLX is a widely used and validated tool for measuring subjective workload across various domains, including health care. Its sensitivity to changes in workload and ease of use make it suitable for evaluating the effect of an AI documentation tool on clinicians’ cognitive load, which correlates with clinician burnout risk.^3^^,^^5^

Providers completed surveys (0-20 scale) after each session (Supplemental Table, available online at https://www.mcpdigitalhealth.org/). Scales were normalized to a 100-point scale and descriptive statistics were used to characterize the study population and NASA-TLX scores. This study was approved as quality improvement by the Kansas University Medical Center institutional review board. We followed Standards for Quality Improvement Reporting Excellence reporting guidelines. The surveys were deidentified to ensure psychological safety in truthfully reporting cognitive load.

## Results

Participants had a median age of 42 years and 11.5 years of practice; 52.5% identified as female. Primary care, medical, and surgical specialties were evenly represented (Table). Use of Abridge was associated with both a significantly lower NASA-TLX composite score—mean difference of 60.7% (221.20 vs 118.20)—and a reduction in each individual subdimension of effort (60.3%, 75.25 vs 40.38), mental demand (64.6%, 74.00 vs 37.88), and temporal demand (57.1%, 72.00 vs 40.00) (all with *P*<.001) (Figure). The observed reductions in composite and subdimension scores indicate that the ambient AI platform substantially lessened cognitive load.

**Table:** Participant Characteristics

Participant demographics	n (%)	Median (IQR)
Age (y)		42 (41-48.5)
Sex		
Male	19 (47.5%)	
Female	21 (52.5%)	
Years in practice		11.5 (8-19.75)
Specialty type		
Primary care	14 (35%)	
Medical subspecialty	14 (35%)	
Surgical specialty	12 (30%)	

IQR, interquartile range.

**Figure:**
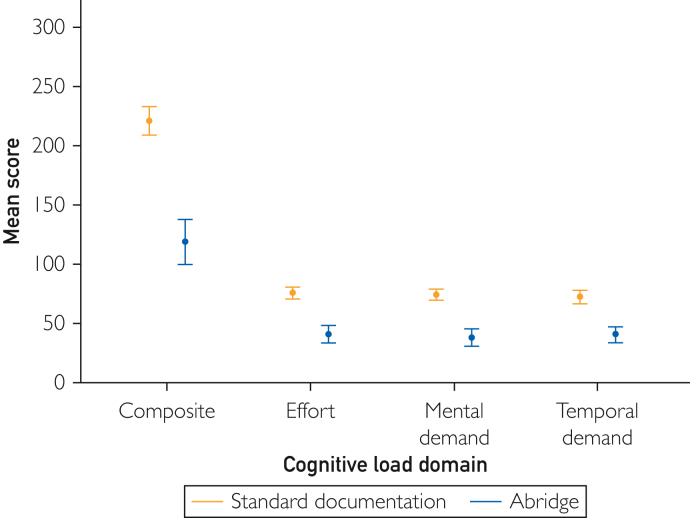
The mean task load scores comparing Abridge and standard documentation. Higher scores indicate a higher cognitive load. Vertical bars indicate 95% confidence intervals.

## Discussion

Our findings report that integrating ambient AI documentation into the participants’ clinical workflow considerably reduced the cognitive burden of documentation across a multispecialty provider population. In the United States, clinicians spend nearly 50% of their time on documentation and administrative work and only 27% on face-to-face patient interaction.^6^ High cognitive load is associated with increased risk of clinician burnout—particularly emotional exhaustion and depersonalization—which adversely affects patient care quality.^5^ Burnout, in turn, has been directly linked to higher rates of medical errors, compromising patient safety.^2^

By alleviating cognitive load, ambient AI tools like Abridge offer a practical solution to mitigate clinician burnout and reduce medical errors. The AI platform may streamline documentation by automating notetaking, allowing clinicians to focus more on patient care. The observed reductions in effort, mental demand, and temporal demand suggest that clinicians can conserve cognitive resources, potentially decreasing feelings of exhaustion and allowing more time for meaningful patient interactions. These findings underscore the importance of interventions that target cognitive load to promote clinician well-being and improve the quality of patient care.

Although limited by a single-center design and convenience sampling, the important reduction in each cognitive load dimension reports potential for substantial, clinical practice-changing impact if offered to all clinicians. Future studies should investigate ambient AI’s impact on clinician burnout and patient safety across larger, more diverse populations of providers and patients and explore potential barriers to widespread adoption. Including various clinical settings and patient demographics will help determine the broader applicability and effectiveness of AI tools in health care.

## Conclusion

Excessive cognitive load in the clinical work environment is an often-overlooked factor contributing to medical errors and compromising patient safety. Our study reports that using an ambient AI platform significantly reduces this burden, addressing a critical issue impacting both health care providers and patients. Implementing such tools is essential for mitigating burnout, decreasing medical errors, and enhancing patient outcomes. This research contributes to the growing body of evidence supporting AI integration in clinical practice.

## Potential Competing Interests

Dr Shah is employed by Abridge AI, Inc, as the Chief Clinical Officer. The other authors declare no competing interests.

## Ethics Statement

This study was approved as quality improvement by the Kansas University Medical Center Institutional Review Board.

## Declaration of generative AI and AI-assisted technologies in the writing process

During the preparation of this work the authors used Microsoft 365 Copilot in order to improve language and readability. After using this tool/service, the authors reviewed and edited the content as needed and take full responsibility for the content of the publication.
