# Supplementary material for: Impact of Ambient Artificial Intelligence Documentation on Cognitive Load
Source: Mayo Clin Proc Digit Health. 2025 Jan 2;3(1):100193. doi: 10.1016/j.mcpdig.2024.100193 (PMC11975833; doi:10.1016/j.mcpdig.2024.100193)
Supplement: Supplemental Table [file mmc1.pdf]

Supplemental Table. Cognitive Load Survey Questions

NASA TLX Question Name	Description
NASA TLX: Effort	On a scale from 0-20, how hard do you have to work to accomplish your level of note-writing performance?
NASA TLX: Mental Demand	On a scale from 0-20, how mentally demanding is it to write your notes?
NASA TLX: Temporal Demand	On a scale from 0-20, how hurried/rushed is the pace of your note writing?
